# What do Women Want? Experiences of Low-Income Women with Postpartum Contraception and Contraceptive Counseling

**DOI:** 10.4172/2376-127X.1000191

**Published:** 2015-09-23

**Authors:** Lynn M Yee, Katherine C Farner, Erin King, Melissa A Simon

**Affiliations:** 1 Department of Obstetrics and Gynecology, Northwestern University, Feinberg School of Medicine, Chicago, IL, United States; 2 Hope Clinic for Women, Granite City, IL, United States

**Keywords:** Postpartum contraception, Family planning, Contraceptive decision making, Health literacy, Health disparities

## Abstract

**Background:**

Contraceptive counseling can increase postpartum contraception use, yet the optimal method and timing for counseling are unknown. The objective was to investigate preferences of underserved pregnant and postpartum women regarding contraception use and counseling.

**Method:**

Surveys regarding contraception experiences and perceptions of contraceptive counseling were conducted with 57 women age 18 and older who were postpartum or antepartum with a previous delivery within 5 years and receiving Medicaid-funded care at an academic medical center. Health literacy was assessed using REALM-7. Responses were analyzed using descriptive statistics.

**Results:**

A majority of women reported unplanned pregnancies (78%). Women using contraception at the time of conception reported “not sure” (30%) and “taken wrong” (30%) as primary reasons for failure. Most subjects had at least a high school level of health literacy (88%), desired to use a postpartum contraceptive method (92%) and had a high self-reported understanding of that method (94%). Most women reported receiving counseling (91%) and stated that the best time for counseling was both before and after childbirth (84%). However, only 60% of subjects intended to use the method they were prescribed at discharge; reasons for changing included side effects (37%), desire for different contraception (23%) and too complicated of a method prescribed (17%).

**Conclusion:**

Women perceived the best timing of contraceptive education to be both antepartum and postpartum. Despite a high frequency of prior contraceptive failure, self-reported understanding of the chosen postpartum contraceptive method was high. Contraception counseling should be tailored to a woman’s perceived needs, with such education occurring frequently and within the context of her health literacy.

## Introduction

It has been well studied that approximately 50% of pregnancies in the United States are unintended [1]. The burden of unintended pregnancy is disproportionately high for African-American and Latina women, as well as higher for women living below the federal poverty line [1]. Unplanned pregnancies are associated with adverse economic and mental health outcomes for mothers and worse health outcomes for children [2]. Improved use of effective contraception, particularly for women with a history of unintended pregnancy, can help reduce the risk of repeat unintended pregnancy. Of all reproductive aged women in the US, 37% are not using any form of contraception, and 20% of those are sexually active yet not seeking to conceive [3]. Further, among women using short-acting reversible contraception (combined hormonal contraceptive methods, injectable hormonal methods, male condoms, withdrawal, and fertility awareness) 12.4% will conceive within a year of “typical use” [4]. Thus, improving the quality of contraceptive counseling and provision may be one way to reduce unintended pregnancy. The antepartum and postpartum periods are potential opportunities for preventive health care in which reliable methods of contraception and counseling on correct use can be addressed.

Focused contraceptive counseling for pregnant and postpartum women significantly increases the likelihood of contraception use [5–7]. However, the best timing and method for such counseling is controversial, and inadequate data have explored women’s experiences of contraceptive counseling. While some studies have found antenatal counseling to be associated with postpartum contraception use [6–8], others have found that antenatal counseling had little effect on contraceptive use or subsequent pregnancy rates [9].Reasons for these differences remain unclear, and in large part may be due to differences in studied populations, variable quality of contraception education provided, and differences in long-term support strategies to ensure maintenance of contraception use. Our group’s previous qualitative work found that women preferred frequent and short provider-initiated contraceptive counseling during the antepartum period [10]. The Cochrane Collaborative Review on contraceptive counseling identified no current standard method, timing of initiation and content of contraceptive counseling, but found interventions with multiple contacts to be promising and warranting further investigation [11]. Guidelines on perinatal care from the American Congress of Obstetricians and Gynecologists (ACOG) recommend contraceptive counseling be a focus during antenatal visits and states that long-acting reversible contraception should be first-line methods; however, recommendations for the means, frequency, timing, and style of such counseling are not described [12].

In addition, a woman’s health literacy may affect her understanding of the contraception counseling interaction. Health literacy is defined as the “capacity to obtain, communicate, process, and understand basic health information and services to make appropriate health decisions” [13]. A recent ACOG Committee Opinion states that low health literacy has an established impact on health outcomes; means by which physicians can mitigate that impact include tailoring speaking skills and health information to the patient [14]. Inadequate health literacy has been associated with both poor understanding of prenatal screening tests and poor maternal health behaviors [15–17]. In the same patient population as this current study, You et al identified higher health literacy to be associated with better patient understanding of preeclampsia [18]. Given this prior work, we hypothesize there may be similar disparities in knowledge regarding contraception [15]. Further, qualitative work has previously found low health literacy to be associated with interview reports of poor contraceptive knowledge and difficulty with contraceptive use [19].

Existing literature on contraception counseling lacks sufficient study of women’s attitudes towards and experiences of postpartum contraception use. Further, inadequate data have examined the relationship between health literacy and contraception. Thus, our goal was to explore women’s perceptions of contraception counseling and their experiences with contraception use, as well as measure health literacy, in attempt to improve the quality of counseling for an underserved population of women. A secondary aimw as to quantify inadequate health literacy in this population and explore the relationships between health literacy and contraception use and understanding.

## Methods

This was an exploratory, cross-sectional survey study conducted at a publicly-funded, hospital-based outpatient clinic staffed by obstetrics and gynecology residents and supervising faculty physicians at a large, academic medical center in Chicago, Illinois. Contraception counseling is a routine component of antenatal care in this setting; in addition, postpartum contraception planning is a central component of postpartum care. Eligible participants included women over the age of 18 who were current or past recipients of antepartum care at this clinic, had birthed their last child at the affiliated hospital and were English-speaking. Subjects were recruited in three groups: (I) immediately postpartum hospital inpatients, (II) outpatients returning for postpartum visit at approximately 6 weeks, (III) outpatients returning for antepartum care at a subsequent pregnancy within 5 years from their last delivery. The final sample included 57 women. Institutional review board approval was obtained for the study through Northwestern University. Women were approached by their health care provider to participate in the study, but surveys were completed in private. Written consent was obtained from each participant.

Using a combination of open-ended, yes/no, “check all that apply” and 5-point Likert scale response questions, the following topics were assessed: (a) General demographics, (b) Contraception attitudes and perceived knowledge, (c) Contraception use in relation to the most recent or current pregnancy, and (d) Receipt and perception of antepartum and postpartum contraceptive counseling in the prior pregnancy. First, participants were asked if they were using birth control at the time of conception (yes/no), and if so, what type. Using a “check all that apply” approach, participants were asked to indicate reasons why they were not taking contraception or why they felt it did not work, if they were using a method. Using a “check all that apply” approach, they were additionally asked to indicate which methods they have taken in the past. Open-ended questions were utilized to ask which methods patients felt were “best” and “worst,” and which they would choose to use again. Personal and family attitudes about contraception were solicited via Likert-scale questions asking about agreement with statements such as “My family wants me to take birth control,” “My partner wants me to take birth control,” and “I want to take birth control now that my pregnancy is over.” Questions asking participants to “check all that apply” also were utilized to assess sources of information/education about contraception, reasons to continue using contraception, and difficulties obtaining contraception. Participants were additionally asked to recall their most recent delivery, and were then asked “yes/no” questions about whether they were counseled about contraception, were provided a method, filled that prescription, and used the method. They were additionally asked to indicate their agreement with statements about their prior counseling, using a “check all that apply” approach. Finally, patients were asked about their understanding of birth control using several Likert-scale questions, such as “I understood how to use the birth control.” Finally, participants were asked several specific questions about the timing of contraception counseling; participants were asked when they received their birth control counseling (at prenatal visits before delivery, in the hospital after delivery, or no counseling) as well as what they felt the best time for such counseling would be (before delivery, after delivery, or both).

In order to briefly assess health literacy, the Rapid Estimate of Adult Literacy in Medicine-7 (REALM-7) was administered by the health care provider [20]. The REALM is a validated screening instrument used by health care providers to assess an adult patient’s ability to read and pronounce common medical words. The original REALM test consists of 125 words from which the raw score can be converted to reading grade level equivalents [21]. The 7-word REALM has been validated on a similar population of women at the same outpatient clinic, and simplifies the screening process [22]. Inadequate health literacy is defined as a score of ≤ 6 correct words, suggestive of a reading level below ninth grade.

All subjects were counseled by the same group of resident and attending physicians who have been trained to provide consistent postpartum contraception counseling on discharge from the hospital as well as during antenatal and postpartum outpatient care. The responses to the survey questions were analyzed using simple descriptive statistics. Chart reviews of the electronic medical record were performed to identify the physician-reported contraceptive method on discharge from most recent delivery. Descriptive statistics were obtained using MiniTab 14 (Minitab Inc., State College, PA).

## Results

Sequential samples of 60 eligible women were approached and 57 consented to participate: 17 group I (immediate postpartum hospital inpatients), 20 group II (outpatients returning for postpartum visit), and 20 group III (outpatients returning for antenatal care with a subsequent pregnancy). Of the 5% of the eligible women who declined to participate, no further information is available due to lack of consent for accessing their records. There were no significant differences between the three groups in any factors analyzed except time since last delivery, which was a mean of 1.4 days for group I, 50.5 days for group II, and 875 days for group III. Thus, all data reported include the three groups together. Socio-demographic information is reported in Table 1. All subjects were insured by Illinois State Medicaid at the time the survey was completed; this program included coverage of all contraceptive methods at no cost to the patient.

### Contraceptive preferences and use

Regarding contraceptive preferences, 92% desired to obtain education on contraception and to use a contraceptive method. They reported their desire for contraception was influenced by their partner (75%) and family (60%). All (100%) responding subjects had used at least one form of birth control before and 81% had used more than one. Of all contraceptive methods, subjects rated depot medroxyprogesterone acetate (DMPA) injection (29%), pills (20%), transdermal patch (17%), and condoms (19%) as “best.” When asked to describe methods they would choose to use again, 22.9% reported they would use DMPA and 22.9% would use pills. Only 5% had used an intrauterine device (IUD). The most commonly noted barrier to obtaining or using contraception was remembering to use the method. Other barriers are described in Table 2. Other than “not getting pregnant,” 33% of participants also chose an additional reason for continuing contraception, such as school, being too young, lack of money, or having job obligations.

Participants were asked about contraceptive use at the time of most recent conception. Although 71% of subjects were using no contraception at the time of becoming pregnant, 78% identified the same pregnancy as unplanned. The remainder of participants reported they desired pregnancy, didn’t mind if they got pregnant or just didn’t want to use contraception. The most common reason for not taking contraception was “too many side effects” (48%). Of the 17 women using contraception at the time of becoming pregnant, the methods used included pills (29%), transdermal patch (29%) and condoms (23%). The most commonly reported reasons for failure were “not sure” (30%) and “taken wrong” (30%). Forty-one percent of these women were restarted on the same method postpartum as the method that they had been on when they conceived.

### Contraceptive education and counseling

When asked about their social network and prior contraceptive education, participants believed 85% of their friends used contraception, with >55% using pills or DMPA. Education about contraception had been obtained from one or more sources including physician (85%), nurse (45%), friends (30%), school (25%), and internet, television or family (16–20%). The best sources of education were felt to be health care providers, including 77% receiving information from a physician and 4.6% from a nurse.

Regarding receipt of contraceptive counseling in the most recent pregnancy, 80% reported receiving antepartum counseling, 30% also received postpartum counseling, while 9% reported receiving no counseling. Eighty-four percent stated the best time for counseling was both before and after childbirth. Median reported amount of counseling received was 20 minutes (range 2 to 180 minutes). Subjects’ agreement with positive and negative aspects of their contraception counseling is outlined in Table 3. Most commonly prescribed postpartum contraceptive methods were pills (30%), IUD (24%), DMPA (16%), and transdermal patch (16%). The vast majority of participants (94%) strongly agreed or agreed that they understood the postpartum contraception method that was prescribed, including when to initiate its use. However, there was 53% agreement between subject’s actual contraception plan (as reported by the patient) and physician-reported contraceptive plan at postpartum discharge. On evaluation of the chosen postpartum method and self-reported level of understanding, women using pills were the most likely to report good understanding of their chosen method (81%) and women using the IUD were least likely to report that they understood the method (33%). Finally, 61% of participants intended to use or did use the contraceptive method prescribed at hospital discharge. The most common reasons for discontinuation were “too many side effects” (37%), desired different contraception (23%), and too complicated of a method prescribed (17%).

### Health literacy and contraception experience

The mean REALM-7 score was 6.35 ± 1.42, with the majority of subjects (88%) having a health literacy rate of high school or above and high education levels (85% ≥ 12 grade).Twelve percent of subjects had inadequate health literacy. While women who agreed that they understood their postpartum contraceptive method had higher REALM-7 scores than those who did not report understanding (6.5 versus 6.2), this difference did not achieve statistical significance. Mean REALM-7 scores were significantly higher for women identifying their current or most recent pregnancy as unplanned versus planned (6.6 versus 5.1, p=0.005), but there was no difference in subjects using contraception at the time of becoming pregnant.

## Discussion

In this group of low-income, minority women receiving public aid for the medical expenses of pregnancy and contraceptive care, there was a higher level of unplanned pregnancy and unprotected intercourse than reported U.S. national averages [23]. Over 90% had received extensive contraceptive counseling during their antenatal clinic visits. All women had positive feedback regarding the contraceptive education they had received, but many felt the timing could be improved. Even though most subjects felt the best time for counseling was both in the antepartum and immediate postpartum period, only 30% reported receiving postpartum counseling, despite standardized hospital discharge instructions that included contraceptive counseling. Our findings highlight the importance of frequent contraceptive counseling in both the antenatal and postpartum periods.

Many studies have examined barriers to contraception in different populations, with reported obstacles including education and literacy [24–26] access, money, insurance, and availability [27–30]. While these obstacles were seen in this population, an important additional focus was perceived side effects and difficulty with use of methods. This echoes other findings regarding lack of contraceptive compliance secondary to method-related reasons [28,31,32]. Both of these factors, perceived side effects and difficulty of use, can be addressed in contraceptive counseling by helping women choose the method they find most convenient while attempting to reduce the risk of side effects they find important. Therefore, we posit that it is essential to comprehend a particular woman’s main obstacles to contraceptive use and to encourage women to discuss their reasons for discontinuation [33]. More importantly, asking women what kind, amount, and depth of information they need regarding contraception allows a provider to best communicate with patients after they have established this understanding of barriers. Further, understanding the patient’s other sources of information may improve the quality of the patient-provider interaction. As much as 20% of participants in our study reported receiving contraception information from other sources, such as the internet and television. In addition to understanding what patients have learned from friends or school education about contraception, we propose that a component of patient counseling about contraception should include eliciting what patients have learned from their media and internet exposure. Particularly in the era of social media use, patients have many potential sources of contraception education, with the possibility of learning both accurate information as well as myths. Providers performing contraceptive counseling may be able to more thoughtfully tailor their health care services to a patient’s needs if they understand her baseline knowledge and preferred sources of information.

Patient-centered contraceptive education that fully incorporates women’s prior experiences and current concerns, while also accounting for her health literacy, allows for an individualized experience that may improve quality of care. While this group exhibited a high prevalence of adequate health literacy and high education levels, prior reports in this population have described lower levels of literacy and education [15]. It is notable that mean REALM-7 scores were significantly higher among women who reported a planned pregnancy, compared to women with unplanned pregnancies; although this was a small study in which a majority of the population experienced unplanned pregnancies, these findings suggest that health literacy may be an important contributory element on the pathway to unintended pregnancy. Additional investigation is warranted to investigate whether these findings persist in broader populations and to understand the manner in which health literacy is associated with pregnancy intendedness. In addition, although the REALM-7 was not associated with contraception use or self-reported understanding, the study was underpowered to detect such differences. It is possible that the REALM-7 is not sufficiently sensitive as a screening tool for contraception assessments; further work is needed to clarify potential relationships between literacy and contraception education understanding and uptake.

In order to provide comprehensive postpartum contraception services, providers need to understand the most effective and preferred periods of time for contraceptive education in the perinatal period. In this population, women perceived the optimal timing for contraceptive education to be both antepartum and postpartum. Current ACOG guidelines recommend that contraceptive counseling be included in antenatal visits but timing is not further specified [12]. Previous literature has demonstrated both antenatal [6–8] and postnatal [6,11] counseling to increase postpartum contraception use. Given the timing preference of this group of women, along with controversy about the optimal period for counseling, we recommend that contraceptive education should occur multiple times throughout pregnancy and postpartum. Our previous work demonstrates that women prefer frequent, brief contraceptive counseling from their providers [10], and therefore this repetition may not only increase contraceptive use, but also patient satisfaction.

This study was a small, exploratory survey study conducted in a single outpatient clinic population. A number of limitations must be considered. Women in this population received care from a discrete group of medical providers at a single institution, and thus may not be generalizable to other populations. In addition, the inclusion of women at multiple time points in their reproductive lives may have contributed to bias due to differences in characteristics between women or in services provided to women at each of these stages of reproductive health care. It is possible that women who were pregnant and reflecting on contraceptive counseling in their prior pregnancy had additional contraceptive education in the interpregnancy interval, which introduces the possibility of recall bias and misclassification bias. Further, it is possible that participant recall of the counseling experience was in accurate (in comparison to provider reports); while ultimately patient recall of counseling is likely the most clinically meaningful aspect of the contraceptive education experience (since counseling that occurred but was not remembered is unlikely to be clinically meaningful), further research should investigate these issues of recall. Moreover, this study did not have the power to detect small differences in item responses and in REALM literacy scores. A larger cohort of women may exhibit significant associations between different factors that were not detected in this study. We also do not have information on the non-responders to the survey. Since these data were self-reported, there is additional possibility for respondent bias and social desirability bias. Finally, this study was performed before subdermal implants were clinically available in our clinic setting, and thus women were not queried about use of this particular highly effective method. However, given the goal of exploring contraceptive experiences in this underserved population, and the secondary goal of generating questions for future investigation, we consider these limitations to be acceptable.

The intent of this study was to examine contraceptive use history and preferences, with the ultimate goal of improving perinatal contraceptive counseling. We have previously qualitatively examined preferences for contraceptive counseling [10], factors resulting in negative counseling encounters [34] and impact of literacy [19] and social network on contraceptive use [35]. With this improved understanding of counseling preferences and experiences, we aim to improve the quality of contraceptive counseling and care provision in this population. Potential interventions to improve the quality of patient-centered care may include simple written or multimedia decision aids that could be tailored to meet individual women’s needs and literacy levels. Such a tool could offer focused education on contraceptive methods while also reviewing instructions, side effects, and warning signs. In this motivated population, tools that augment individualized counseling may be an effective method for improving contraceptive knowledge and optimizing contraception uptake and retention. In a review of compliance with oral contraceptive use, Rosenberg et al concluded “good compliance has been linked with patient satisfaction with the clinician, the absence of certain side effects… and reading information distributed” [36]. Ultimately, patient-centered care that addresses the highly individualized topic of contraceptive use in a thoughtful and comprehensive manner, with potential use of supplementary education tools, may address a number of the issues identified in this pilot survey of underserved women’s experiences with contraception.

## Figures and Tables

**Table 1:** Participant demographics.

	% or mean (SD) N=57

Age (years)	26.7 ± 5.1

Race	
African American	63.6%
Hispanic	23.6%
Asian	9.1%
White	3.6%

Education	
Less than high school graduate	14.1%
High school graduate	19.3%
Some college or greater	66.0%

Employment >35 hours per week	70.2%

Current Relationship	
Committed relationship	53.6%
Married	19.6%
Single	26.8%

Father of baby involved	94.6%

Gravidity	3.3 ± 2.0

Parity	2.2 ± 1.3

Age at first intercourse (years)	16.6 ± 2.8

**Table 2:** Participant-reported difficulties in obtaining or continuing contraception.

	% responding in agreement N=57
Easy to forget	42%
Difficult to use	18%
Lack of insurance	18%
No access to contraception	16%
Insufficient money	8%
Unavailability	8%
Does not fit in lifestyle	6%
Annoyance	6%
Not caring about it	6%
Partner does not want subject to take	4%
Pressure from family	2%
Embarrassed to take	0 (0%)
Religious factors	0 (0%)

**Table 3:** Perceptions of antepartum/immediate postpartum contraceptive counseling.

	% responding in agreement N=57
I received plenty of counseling	70%
My doctor took time to counsel me	68%
I was given several contraceptive options	57%
The counseling helped me understand what I needed to know about birth control	51%
Counseling helped me make a good decision	38%
I wish I was given more options of birth control	12%
I wish I received more counseling	2%
I received too much counseling	2%
I did not receive enough counseling	0%
I was forced to make a decision about birth control without a lot of information	0%
The counseling was complicated	0%
My doctor was too busy for good counseling	0%
